# Implementation of a high sensitivity cardiac troponin I assay and risk of myocardial infarction or death at five years: observational analysis of a stepped wedge, cluster randomised controlled trial

**DOI:** 10.1136/bmj-2023-075009

**Published:** 2023-11-28

**Authors:** Kuan Ken Lee, Dimitrios Doudesis, Amy V Ferry, Andrew R Chapman, Dorien M Kimenai, Takeshi Fujisawa, Anda Bularga, Matthew T H Lowry, Caelan Taggart, Stacey Schulberg, Ryan Wereski, Chris Tuck, Fiona E Strachan, David E Newby, Atul Anand, Anoop S V Shah, Nicholas L Mills, High-STEACS Investigators, Stephanie Barker, Jennifer Blades, Konstantin Georgiev, Jeremy Leung, Ziwen Li, Lynn McKinlay, Michael McDermott, Jasper Boeddinghaus, Jean McPherson, Filip Mendusic, Andrew Sorbie, Grace Souter, Daniel Perez Vicencio, Yiqing Wang, Kelly Williams, Keith A A Fox, Colin Berry, Simon Walker, Christopher J Weir, Ian Ford, Alasdair Gray, Paul O Collinson, Fred S Apple, Alan Reid, Anne Cruikshank, Iain Findlay, Shannon Amoils, David A McAllister, Donogh Maguire, Jennifer Stevens, John Norrie, Jack Andrews, Philip D Adamson, Alastair Moss, Mohamed S Anwar, John Hung, Jonathan Malo, Colin M Fischbacher, Bernard L Croal, Stephen J Leslie, Catriona Keerie, Richard A Parker, Allan Walker, Ronnie Harkess, Tony Wackett, Roma Armstrong, Laura Stirling, Claire MacDonald, Imran Sadat, Frank Finlay, Heather Charles, Pamela Linksted, Stephen Young, Bill Alexander, Chris Duncan

**Affiliations:** 1 British Heart Foundation (BHF) Centre for Cardiovascular Science, University of Edinburgh, Edinburgh EH16 4SA, UK; 2 Usher Institute, University of Edinburgh, Edinburgh, UK; 3 London School of Hygiene and Tropical Medicine, London, UK

## Abstract

**Objective:**

To evaluate the impact of implementing a high sensitivity assay for cardiac troponin I on long term outcomes in patients with suspected acute coronary syndrome.

**Design:**

Secondary observational analysis of a stepped wedge, cluster randomised controlled trial.

**Setting:**

10 secondary and tertiary care centres in Scotland, UK.

**Participants:**

48 282 consecutive patients with suspected acute coronary syndrome. Myocardial injury was defined as any high sensitivity assay result for cardiac troponin I >99th centile of 16 ng/L in women and 34 ng/L in men.

**Intervention:**

Hospital sites were randomly allocated to either early (n=5 hospitals) or late (n=5 hospitals) implementation of a high sensitivity cardiac troponin I assay with sex specific diagnostic thresholds.

**Main outcome measure:**

The main outcome was myocardial infarction or death at five years.

**Results:**

10 360 patients had cardiac troponin concentrations greater than the 99th centile, of whom 1771 (17.1%) were reclassified by the high sensitivity assay. The five year incidence of subsequent myocardial infarction or death before and after implementation of the high sensitivity assay was 29.4% (5588/18 978) *v* 25.9% (7591/29 304), respectively, in all patients (adjusted hazard ratio 0.97, 95% confidence interval 0.93 to 1.01), and 63.0% (456/720) *v* 53.9% (567/1051), respectively, in those reclassified by the high sensitivity assay (0.82, 0.72 to 0.94). After implementation of the high sensitivity assay, a reduction in subsequent myocardial infarction or death was observed in patients with non-ischaemic myocardial injury (0.83, 0.75 to 0.91) but not in those with type 1 or type 2 myocardial infarction (0.92, 0.83 to 1.01 and 0.98, 0.84 to 1.14).

**Conclusions:**

Implementation of a high sensitivity cardiac troponin I assay in the assessment of patients with suspected acute coronary syndrome was associated with a reduced risk of subsequent myocardial infarction or death at five years in those reclassified by the high sensitivity assay. Improvements in outcome were greatest in patients with non-ischaemic myocardial injury, suggesting a broader benefit beyond the identification of myocardial infarction.

**Trial registration:**

ClinicalTrials.gov NCT01852123.

## Introduction

High sensitivity cardiac troponin assays utilise improved precision at very low concentrations to improve the diagnosis and risk stratification of patients with suspected acute coronary syndrome.^1^ ^2^ As such, the Universal Definition of Myocardial Infarction recommends the use of high sensitivity cardiac troponin assays with sex specific 99th centile thresholds for the diagnosis of myocardial injury and infarction.^3^ These recommendations are now increasingly adopted worldwide, but their impact on outcomes remains uncertain.^4^

The High-Sensitivity Troponin in the Evaluation of patients with suspected Acute Coronary Syndrome (High-STEACS) trial was a randomised controlled trial to evaluate the impact of implementing these recommendations from the Universal Definition of Myocardial Infarction.^5^ We previously found that implementation of a high sensitivity cardiac troponin I assay with a sex specific 99th centile as the diagnostic threshold identified more patients with myocardial injury and infarction and increased the provision of evidence based treatments, but implementation of the assay did not significantly reduce subsequent cardiac events at one year.^5^ Given that these evidence based treatments have been shown to have long term benefits, we hypothesised that changes in care after implementation of high sensitivity testing for cardiac troponin could reduce the risk of myocardial infarction or death beyond one year.^6^ ^7^

In this secondary observational analysis of the High-STEACS trial, we aimed to determine whether implementation of a high sensitivity cardiac troponin I assay could reduce myocardial infarction or death at five years in patients presenting with suspected acute coronary syndrome.

## Methods

### Trial design and participants

We performed a secondary observational analysis of the High-STEACS trial. High-STEACS was a stepped wedge, cluster randomised controlled trial across 10 secondary and tertiary care hospitals in Scotland, UK. Consecutive patients presenting to the emergency department with suspected acute coronary syndrome were screened by the attending clinician using an electronic form for cardiac troponin testing that was embedded within the clinical care pathway. Patients with suspected acute coronary syndrome and with cardiac troponin levels measured using the standard care and trial assays were eligible for inclusion. We excluded those who had already been admitted to hospital during the trial period or who were not resident in Scotland (see supplementary eText 1).

### Randomisation

Hospital sites were the unit of randomisation in this trial. Cluster randomisation was necessary to avoid the risk of clinical error from the concurrent reporting of different troponin thresholds and assays. During standard care, all sites initially reported cardiac troponin levels measured using the contemporary cardiac troponin assay, along with corresponding diagnostic thresholds, for at least six months. During the randomisation period, sites were randomly allocated to either early or late implementation of the high sensitivity cardiac troponin assay with sex specific 99th centile thresholds.

### Trial procedures and blinding

Patients underwent cardiac troponin testing at presentation and six or 12 hours after the onset of symptoms at the discretion of the attending clinician in accordance with contemporaneous national and international practice guidelines during the conduct of the trial.^8^ ^9^ Throughout the trial, cardiac troponin was measured simultaneously in all patients with a contemporary (standard care) assay and a high sensitivity (implementation) assay using plasma that was surplus to clinical requirements. During standard care, attending clinicians were blinded to the results of the high sensitivity assay, and the contemporary assay was used to guide care. Conversely, after implementation of the high sensitivity assay, the clinicians were blinded to the results of the contemporary assay, and the high sensitivity assay with sex specific 99th centile thresholds was used to guide care.

The standard care assay was a contemporary cardiac troponin I assay (Abbott Laboratories; Abbott Park, IL), which has a coefficient of variation of <10% at 0.04 µg/L at seven hospital sites and 0.05 µg/L at three hospital sites. During standard care, only results above these thresholds were reported. The high sensitivity assay was the ARCHITECT_STAT_ high-sensitive troponin I assay (Abbott Laboratories). This assay has a coefficient of variation of <10% at 4.7 ng/L and a 99th centile upper reference limit of 34 ng/L in men and 16 ng/L in women.^10^ During implementation of the high sensitivity assay, all results above the limit of detection of 1.2 were reported in ng/L.

### Diagnostic adjudication

The diagnosis for all patients with high sensitivity troponin I assay levels above the sex specific 99th centile during the index attendance was adjudicated in accordance with the third Universal Definition of Myocardial Infarction.^11^ Two doctors blinded to study phase independently adjudicated the diagnosis after reviewing all clinical information in the patient’s electronic health record. A third reviewer resolved any discordant diagnoses. Type 1 myocardial infarction was defined as myocardial necrosis (any high sensitivity troponin I assay level above the sex specific 99th centile with a rise or fall in high sensitivity troponin I level where serial testing was performed) in the context of a presentation with symptoms or signs of myocardial ischaemia that was consistent with an acute coronary syndrome. Type 2 myocardial infarction was defined as myocardial necrosis with symptoms or signs of myocardial ischaemia where there was evidence of increased myocardial oxygen demand or decreased supply secondary to an alternative condition without evidence of acute atherothrombosis. Patients with high sensitivity troponin I assay levels above the sex specific 99th centile without symptoms or signs of myocardial ischaemia were classified as having non-ischaemic myocardial injury. The cause of non-ischaemic myocardial injury was recorded prospectively and stratified as cardiac or non-cardiac (see supplementary eText 1).

### Trial outcomes

The Scottish Morbidity Record 01 and National Records of Scotland registries are audited regularly for accuracy and were used to ensure complete follow-up for the trial population.^12^ ^13^ ^14^ The primary outcome of the trial was subsequent type 1 or type 4b myocardial infarction or cardiovascular death within one year of the index attendance. However, because no formal event adjudication was performed after one year and events were classified using diagnostic codes, our prespecified primary outcome was any myocardial infarction or all cause death at five years. Secondary outcomes were any myocardial infarction, coronary revascularisation, all cause death, cardiovascular death, cardiac death, and hospital admission for heart failure, ischaemic stroke, and major haemorrhage (see supplementary eText 1).

### Statistical analysis

The study population was stratified by the maximum cardiac troponin level on serial testing. Patients reclassified by the high sensitivity assay were defined as those with cardiac troponin I levels above the sex specific 99th centile but below the contemporary assay diagnostic threshold. Patients with high sensitivity troponin I assay levels below the sex specific 99th centile were classified as having no myocardial injury, whereas those already identified by the contemporary assay were those with any cardiac troponin I level greater than the diagnostic threshold of this assay.

Using a Cox proportional hazards regression model, we compared outcomes before and after implementation of the high sensitivity assay in all patients and in those reclassified by the high sensitivity assay. The model adjusted for hospital site, season (spring, summer, autumn, with winter as the reference category), patient age, sex, comorbidities (diabetes mellitus, previous myocardial infarction, and previous cerebrovascular disease), and an indicator variable for whether the high sensitivity assay had or had not been implemented. Hospital site was fitted as a random effect, and age, sex, and comorbidities were included as fixed patient level covariates in the model. We performed a subgroup analysis stratified by the index diagnosis according to the Universal Definition of Myocardial Infarction^3^ ^15^ and a sensitivity analysis restricted to the randomisation period of the trial to evaluate potential for confounding by secular trends. All analyses were performed in R version 4.1.3.

### Patient and public involvement

The trial steering committee included patient and lay representatives who were involved in the design and conduct of this trial.

## Results

Between 10 June 2013 and 3 March 2016 we enrolled 48 282 consecutive patients with suspected acute coronary syndrome, 22 565 (46.7%) of whom were women. Mean age was 61 years (standard deviation 17 years). Overall, 18 978 (39.3%) patients were enrolled during standard care and 29 304 (60.7%) after implementation of the high sensitivity assay (table 1, also see supplementary eTable 1 and eFigure 1). During the index attendance, 10 360 patients had a high sensitivity troponin I assay level above the sex specific 99th centile, with 17.1% (1771/10 360) reclassified by the high sensitivity assay and 82.9% (8589/10 360) identified by the contemporary assay.

**Table 1:** Baseline characteristics of trial participants stratified by peak cardiac troponin I level and study phase (standard care and after implementation of the high sensitivity assay). Values are number (percentage) unless stated otherwise

Characteristics	Overall			No myocardial injury			Myocardial injury or infarction				
							Reclassified by high sensitivity assay			Identified by contemporary assay	
	Standard care (n=18 978)	After implementation (n=29 304)		Standard care (n=14 862)	After implementation (n=23 060)		Standard care (n=720)	After implementation (n=1051)		Standard care (n=3396)	After implementation (n=5193)
Median (IQR) age (years)	62 (50-76)	60 (4974)		59 (47-73)	57 (46-71)		78 (66-85)	78 (66-85)		73 (61-83)	71 (59-81)
Women	9114 (48)	13 448 (46)		7042 (47)	10 529 (46)		612 (85)	858 (82)		1460 (43)	2061 (40)
Presenting symptoms*:											
Chest pain	10 693 (80)	23 847 (81)		8677 (83)	19 414 (84)		373 (68)	701 (67)		1643 (68)	3732 (72)
Dyspnoea	748 (6)	1427 (5)		383 (4)	724 (3)		66 (12)	136 (13)		299 (12)	567 (11)
Palpitations	386 (3)	883 (3)		291 (3)	700 (3)		19 (3)	53 (5)		76 (3)	130 (3)
Syncope	829 (6)	1666 (6)		567 (5)	1242 (5)		42 (8)	83 (8)		220 (9)	341 (7)
Other	720 (5)	1468 (5)		490 (5)	968 (4)		50 (9)	78 (7)		180 (7)	422 (8)
Medical history:											
Myocardial infarction	1969 (10)	2245 (8)		1353 (9)	1482 (6)		97 (13)	122 (12)		519 (15)	641 (12)
Cerebrovascular disease	1332 (7)	1617 (6)		877 (6)	1038 (5)		88 (12)	122 (12)		367 (11)	457 (9)
Diabetes mellitus	1572 (8)	1946 (7)		961 (6)	1079 (5)		95 (13)	123 (12)		516 (15)	744 (14)
Previous revascularisation:											
PCI	1581 (8)	2101 (7)		1204 (8)	1540 (7)		66 (9)	89 (8)		311 (9)	472 (9)
CABG	349 (2)	433 (1)		238 (2)	296 (1)		18 (3)	22 (2)		93 (3)	115 (2)
Drugs at presentation:											
Aspirin	5840 (31)	7323 (25)		4231 (28)	5231 (23)		300 (42)	368 (35)		1309 (39)	1724 (33)
Dual antiplatelet therapy†	843 (4)	762 (3)		583 (4)	520 (2)		41 (6)	47 (4)		219 (6)	195 (4)
Statin	8191 (43)	11 175 (38)		5994 (40)	8112 (35)		403 (56)	557 (53)		1794 (53)	2506 (48)
ACE inhibitor or ARB	6449 (34)	9169 (31)		4681 (31)	6604 (29)		317 (44)	445 (42)		1451 (43)	2120 (41)
β blocker	5739 (30)	7434 (25)		4183 (28)	5383 (23)		285 (40)	373 (35)		1271 (37)	1678 (32)
Oral anticoagulant‡	1411 (7)	1842 (6)		938 (6)	1220 (5)		94 (13)	144 (14)		379 (11)	478 (9)
Electrocardiogram§:											
Normal	-	-		-	-		77 (31)	255 (38)		209 (26)	735 (26)
Myocardial ischaemia	-	-		-	-		35 (14)	91 (14)		316 (39)	1163 (42)
ST segment elevation	-	-		-	-		7 (3)	17 (3)		137 (17)	576 (21)
ST segment depression	-	-		-	-		26 (11)	57 (9)		167 (21)	551 (20)
Left bundle branch block	-	-		-	-		20 (8)	60 (9)		77 (10)	177 (6)
T wave inversion	-	-		-	-		35 (14)	78 (12)		139 (17)	421 (15)
Physiological variables:											
Mean (SD) heart rate (beats/min)	-	-		-	-		85 (28)	87 (27)		88 (29)	84 (26)
Mean (SD) systolic blood pressure (mm Hg)	-	-		-	-		144 (28)	144 (28)		136 (30)	138 (30)
Haematology and clinical chemistry:											
Mean (SD) haemoglobin (g/L)	135 (22)	136 (21)		137 (20)	138 (20)		126 (22)	124 (22)		132 (26)	133 (25)
eGFR (mL/min/1.73m^2^)	55 (12)	53 (13)		56 (10)	55 (11)		47 (15)	48 (15)		48 (16)	47 (16)
Median (IQR) peak cardiac troponin (ng/L)	4 (2-16)	4 (2-16)		3 (1-6)	3 (1-6)		25 (20-35)	27 (21-38)		215 (62-1566)	398 (87-3584)

ACE=angiotensin converting enzyme; ARB=angiotensin receptor blockers; CABG=coronary artery bypass grafting; eGFR=estimated glomerular filtration rate; IQR=interquartile range; PCI=percutaneous coronary intervention; SD=standard deviation.

* Missing in 4466 (12%) patients.

† Combination of two drugs from aspirin, clopidogrel, prasugrel, or ticagrelor.

‡ Includes warfarin or direct oral anticoagulants.

§ Electrocardiographic findings and physiological variables only reported for those with elevated cardiac troponin levels.

The panel was able to adjudicate the index diagnosis in 88.0% (9115/10 360) of patients with cardiac troponin levels above the sex specific 99th centile. For type 1 myocardial infarction, discordant diagnoses between the first and second adjudicators occurred in 11.6% of all patients (κ=0.76), while across all diagnoses, discordance occurred in 21.7% of patients (κ=0.70). Type 1 myocardial infarction was diagnosed in 55.2% (5028/9115) of patients, type 2 myocardial infarction in 13.8% (1260/9115), and non-ischaemic myocardial injury in 30.8% (2810/9115). The underlying cause of non-ischaemic myocardial injury was cardiac in 47.8% (1335/2792) of patients and non-cardiac in the remainder. Compared with those identified by the contemporary assay, patients reclassified by the high sensitivity assay were more likely to have non-ischaemic myocardial injury (51.3% *v* 26.6%) and less likely to have type 1 myocardial infarction (33.2% *v* 59.7%; P<0.001 for both).

Duration of hospital stay was longer after implementation of the high sensitivity assay compared with standard care in reclassified patients (median 51 hours (interquartile range 20-134 hours) *v* 21 (4-101), P<0.001) but was shorter in those without myocardial injury (4 (3-20) *v* 7 (3-24), P<0.001). Patients reclassified by the high sensitivity assay were more likely to receive dual antiplatelet therapy, receive statin treatment, and undergo invasive coronary angiography after implementation of the high sensitivity assay, but the rate of coronary revascularisation did not differ (see supplementary eTable 2).

Compared with patients who received standard care, patients with type 1 myocardial infarction were more likely to receive dual antiplatelet therapy (62.2% *v* 55.3%), undergo invasive coronary angiography (65.0% *v* 56.7%), and undergo coronary revascularisation (48.2% *v* 38.7%) after implementation of the high sensitivity assay, whereas these interventions for acute coronary syndrome did not differ in those with type 2 myocardial infarction or non-ischaemic myocardial injury (see supplementary eTable 3).

**Fig 1:**
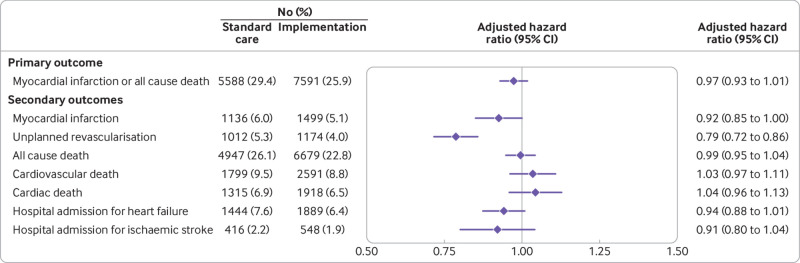
Outcomes at five years in all patients before and after implementation of a high sensitivity cardiac troponin I assay. Forest plot shows the number (percentage) of patients in the standard care and implementation phases, and the hazard ratios for implementation versus standard care for the primary and secondary outcomes adjusted for hospital site, season, age, sex, and comorbidities (diabetes mellitus, previous myocardial infarction, and previous cerebrovascular disease). Whiskers represent 95% confidence intervals

At five years, 27.3% (13 179/48 282) of patients had a subsequent myocardial infarction or death from any cause. In all patients with suspected acute coronary syndrome, the primary outcome of subsequent myocardial infarction or death at five years occurred in 29.4% (5588/18 978) of patients before and 25.9% (7591/29 304) of patients after implementation of the high sensitivity assay (table 2), with an adjusted hazard ratio of 0.97 (95% confidence interval 0.93 to 1.01) (fig 1). The assumptions of the Cox proportional hazards regression model were satisfied. The five year incidence of subsequent myocardial infarction or all cause death in those reclassified by the high sensitivity assay was 63.0% (456/720) in standard care and 53.9% (567/1051) after implementation of the high sensitivity assay (0.82 (0.72 to 0.94)) (see supplementary eFigure 2). Similar findings were observed in a sensitivity analysis restricted to the randomisation phase of the trial, where both assays were used across sites in parallel to guide care (0.87 (0.78 to 0.97) and 0.76 (0.64 to 0.91) for all patients and those reclassified, respectively).

**Table 2:** Outcomes after five years in participants stratified by peak cardiac troponin I level and study phase (standard care or after implementation of the high sensitivity assay). Values are number (percentage)

	Overall			No myocardial injury			Myocardial injury or infarction				
							Reclassified by high sensitivity assay			Identified by contemporary assay	
	Standard care (n=18 978)	After implementation (n=29 304)		Standard care (n=14 862)	After implementation (n=23 060)		Standard care (n=720)	After implementation (1051)		Standard care (n=3396)	After implementation (n=5193)
Myocardial infarction or all cause death	5588 (29)	7591 (26)		3194 (21)	4361 (19)		456 (63)	567 (54)		1938 (57)	2663 (51)
Myocardial infarction	1136 (6)	1499 (5)		584 (4)	757 (3)		103 (14)	113 (11)		449 (13)	629 (12)
Coronary revascularisation	1012 (5)	1174 (4)		729 (5)	808 (4)		41 (6)	45 (4)		242 (7)	321 (6)
Death:											
All cause	4947 (26)	6679 (23)		2804 (19)	3837 (17)		416 (58)	501 (48)		1727 (51)	2341 (45)
Cardiovascular	1799 (9)	2591 (9)		841 (6)	1186 (5)		170 (24)	222 (21)		788 (23)	1183 (23)
Cardiac	1315 (7)	1918 (7)		569 (4)	803 (3)		118 (16)	167 (16)		628 (18)	948 (18)
Reason for hospital admission:											
Heart failure	1444 (8)	1889 (6)		744 (5)	931 (4)		144 (20)	198 (19)		556 (16)	760 (15)
Ischaemic stroke	416 (2)	548 (2)		271 (2)	351 (2)		34 (5)	38 (4)		111 (3)	159 (3)
Major haemorrhage*	318 (2)	381 (1)		220 (1)	260 (1)		19 (3)	26 (2)		79 (2)	95 (2)

* Defined as Bleeding Academic Research Consortium type 3 or 5.

**Fig 2:**
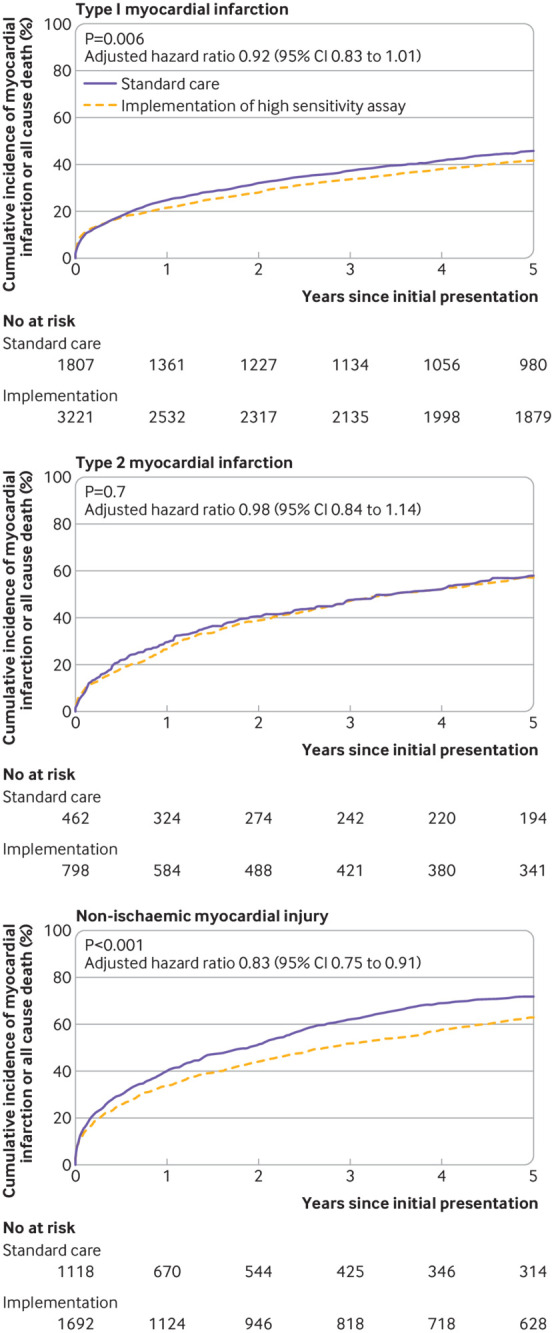
Cumulative incidence of myocardial infarction or all cause death in all patients stratified by index diagnosis of type 1 myocardial infarction (log rank test P=0.006), type 2 myocardial infarction (log rank test P=0.70), and non-ischaemic myocardial injury (log rank test P<0.001) during standard care and following implementation of a high sensitivity cardiac troponin I assay

After implementation of the high sensitivity assay, a reduction in subsequent myocardial infarction or death was observed in patients with non-ischaemic myocardial injury (0.83, 0.75 to 0.91) but not in those with type 1 or type 2 myocardial infarction (0.92, 0.83 to 1.01 and 0.98, 0.84 to 1.14), respectively (fig 2, also see supplementary eTable 5 and eFigure 3). Similar findings were observed in a subgroup analysis stratified by index diagnosis adjudicated in accordance with the fourth Universal Definition of Myocardial Infarction (see supplementary eTable 4). In patients with non-ischaemic myocardial injury, the reduction in subsequent myocardial infarction or death at five years was greater in those with a cardiac cause of non-ischaemic myocardial injury (0.69 (0.60 to 0.80)) compared to those with a non-cardiac cause (0.95 (0.83 to 1.09)) (see supplementary eFigure 4).

## Discussion

In the primary report from the High-STEACS trial, we found that implementation of a high sensitivity cardiac troponin assay with sex specific 99th centile thresholds reclassified nearly one in five patients with myocardial injury and increase the provision of evidence based treatments.^5^ In this secondary analysis of longer term follow-up, we report an association between implementation of the high sensitivity assay and fewer subsequent myocardial infarctions or deaths at five years in those patients reclassified using the high sensitivity assay. The improvement in outcomes was greater in patients with an index diagnosis of non-ischaemic myocardial injury compared to those with type 1 or type 2 myocardial infarction.

Our original hypothesis was that the implementation of the high sensitivity assay and the use of a lower diagnostic threshold would identify patients with a missed diagnosis of myocardial infarction using less sensitive contemporary troponin assays, and that recognition of these patients would result in better care and outcomes.^16^ ^17^ Consistent with other studies, however, we observed that only a few patients reclassified by the high sensitivity assay had type 1 myocardial infarction, and that most had non-ischaemic myocardial injury.^18^ ^19^ ^20^ ^21^ ^22^ Indeed one of the main concerns that has limited uptake of high sensitivity cardiac troponin testing in clinical practice is that lower diagnostic thresholds will reduce the specificity of cardiac troponin for type 1 myocardial infarction, which could result in misdiagnosis and unnecessary investigation or treatment.^23^ We found no evidence of unnecessary treatment for acute coronary syndrome or harm in patients identified as having non-ischaemic myocardial injury. On the contrary, we observed that improvements in outcomes at five years were greatest in those patients with an index diagnosis of non-ischaemic myocardial injury.

### Comparison with other studies

Although previous randomised trials have shown that implementation of high sensitivity cardiac troponin testing as part of an early diagnostic pathway reduces hospital admissions and the duration of stay in patients without myocardial injury,^1^ ^24^ we found that use of a high sensitivity assay could also improve outcomes in patients with evidence of myocardial injury. Although our observation that the greatest benefit was in patients with non-ischaemic myocardial injury was unexpected, it is not only plausible but intuitive and consistent with a growing body of evidence showing the value of high sensitivity cardiac troponin testing in conditions other than myocardial infarction.^25^ ^26^ ^27^ ^28^

### Implications for practice and future research

In practice, myocardial infarction is differentiated from other mechanisms of myocardial injury by the presence of symptoms or signs of myocardial ischaemia, or by definitive cardiac imaging. Patients with an adjudicated diagnosis of myocardial infarction who were reclassified by the high sensitivity assay in standard care, but with the results concealed, may already have been recognised as being at increased risk or in need of further assessment owing to symptoms or electrocardiographic findings. As such, there was perhaps less to gain from recognising these patients as having elevated cardiac troponin levels after high sensitivity testing, unlike those with non-ischaemic myocardial injury that may be silent and only identified by troponin testing.

Cardiac troponin is now widely recognised as a powerful independent prognostic marker in patients without type 1 myocardial infarction across a diverse range of acute cardiac and non-cardiac conditions, such as sepsis, renal failure, pulmonary embolism, heart failure, after surgical interventions, and, more recently, in patients with SARS-CoV-2 infection.^25^ ^26^ ^27^ ^28^ ^29^ ^30^ ^31^ As such, clinicians are perhaps more likely to admit patients with an elevated cardiac troponin level even after the identification of an alternative explanation for their presentation. Consistent with this, we observed that the duration of stay doubled in patients reclassified by the high sensitivity assay. Multiple studies have shown that elevated cardiac troponin concentrations are common in cardiac conditions such as chronic heart failure, stable coronary artery disease, and valvular heart disease.^32^ ^33^ ^34^ Improved recognition of these conditions after implementation of high sensitivity testing could explain why the greatest reduction in events was observed in those with a cardiac cause of non-ischaemic myocardial injury. Longer hospital stays may have facilitated additional specialist review, and further investigation, such as echocardiography, after myocardial infarction was excluded. Although these investigations were not recorded in the trial database, which captured information on the management of acute coronary syndrome, the diagnosis and subsequent follow-up of other newly recognised cardiac conditions could impact long term outcomes.

### Limitations of this study

We acknowledge several important limitations. Firstly, we were not able to adjudicate outcome events beyond the first year of follow-up. Our outcome measure of any subsequent myocardial infarction or death from any cause was prespecified as it is less susceptible to misclassification bias through use of diagnostic coding.^12^ ^13^ ^14^ Arguably, coded hospital admissions are more meaningful than those defined by adjudication as these are the events that matter to the healthcare system.^35^ Secondly, our pragmatic design meant that we had to accept some flexibility in the date of implementation of the high sensitivity assay to accommodate the shared laboratory services. This resulted in long before and after periods that may be more susceptible to the influence of secular trends. However, we observed similar findings in our sensitivity analysis restricted to the randomisation phase when both the standard care and the high sensitivity assay were running in parallel. Thirdly, we were unable to evaluate the impact of implementing high sensitivity cardiac troponin testing across different ethnicities because less than 5% of our study population were of non-white ethnicity. However, a recent study did not identify any difference in troponin thresholds across different ethnic groups in a more diverse US population^36^; therefore, we do not believe that effectiveness of high sensitivity cardiac troponin assays is likely to differ. Finally, our trial captured only a limited number of investigations and treatments for myocardial infarction during the index attendance. Further research is required to understand how implementation influenced the management and outcomes of patients with non-ischaemic myocardial injury.

### Conclusions

In patients with suspected acute coronary syndrome, implementation of a high sensitivity cardiac troponin assay reduced subsequent myocardial infarction or death at five years in those reclassified by the high sensitivity assay. Improvements in outcome were greatest in patients with a diagnosis of non-ischaemic myocardial injury, suggesting cardiac troponin testing may have benefits beyond the identification of myocardial infarction.

**What is already known on this topic**

The introduction of high sensitivity cardiac troponin testing has identified more patients with myocardial injury and infarction than previous generations of cardiac troponin assays, but whether this has improved outcomes remains uncertain
In the primary report from this trial, it was found that implementation of a high sensitivity troponin I assay reclassified nearly one in five patients with myocardial injury or infarction and increased the provision of evidence based treatments

**What this study adds**

Implementation of a high sensitivity cardiac troponin assay with sex specific 99th centile thresholds resulted in fewer subsequent myocardial infarctions or deaths at five years in patients reclassified by the high sensitivity assay
The improvement in outcomes was greater in patients with an index diagnosis of non-ischaemic myocardial injury compared to those with type 1 or type 2 myocardial infarction

## Data Availability

The High-STEACS trial makes use of several routine electronic healthcare data sources that are linked, deidentified, and held in a national safe haven, which is accessible by approved individuals who have undertaken the necessary governance training. Summary data can be made available upon request to the corresponding author.

## Web extra

Extra material supplied by authors

Supplementary information: Supplementary eText 1, eTables 1-5, and eFigures 1-4

## Notes

Contributors: KKL and DD contributed equally. KKL, ASVS, and NLM conceived and designed the study. The High-STEACS Investigators acquired the data. KKL and DD performed the analysis. KKL, DD, AVF, ARC, DK, TF, AB, ML, CT, SS, RW, CT, FES, DEN, AA, ASVS, and NLM interpreted the data. KKL and NLM drafted the manuscript. KKL, DD, AVF, ARC, DK, TF, AB, ML, CT, SS, RW, CT, FES, DEN, AA, ASVS, and NLM revised the manuscript critically for important intellectual content. All authors provided their final approval of the version to be published. All authors are accountable for the work. KKL, DD, and NLM are the study guarantors. The corresponding author attests that all listed authors meet authorship criteria and that no others meeting the criteria have been omitted.

The lead author (KKL) affirms that the manuscript is an honest, accurate, and transparent account of the study being reported; that no important aspects of the study have been omitted; and that any discrepancies from the study as planned have been explained.

Dissemination to participants and related patient and public communities: Findings of this study will be shared with clinicians and patients through national and international cardiology conferences, and through social media platforms. A plain language summary will also be disseminated through a press release.

Provenance and peer review: Not commissioned; externally peer reviewed.
