# Supplementary material for: Implementation of a high sensitivity cardiac troponin I assay and risk of myocardial infarction or death at five years: observational analysis of a stepped wedge, cluster randomised controlled trial
Source: BMJ. 2023 Nov 28;383:e075009. doi: 10.1136/bmj-2023-075009 (PMC10680066; doi:10.1136/bmj-2023-075009)

SUPPLEMENT

Implementation of a high sensitivity cardiac troponin I assay and risk of myocardial infarction or death at five years: observational analysis of a stepped wedge, cluster randomised controlled trial

Kuan Ken Lee, M.D.,[1*] Dimitrios Doudesis, Ph.D.,[1, 2*] Amy V. Ferry, Ph.D.,[1] Andrew R. Chapman, M.D.,[1] Dorien M. Kimenai, Ph.D.,[1] Takeshi Fujisawa, Ph.D.,[1] Anda Bularga, M.D.,[1] Matthew T.H. Lowry, M.D.,[1] Caelan Taggart, M.D.,[1] Stacey Schulberg, M.Sc.,[1] Ryan Wereski, M.D.,[1] Chris Tuck, B.Sc.,[1] Fiona E. Strachan, Ph.D.,[2] David E. Newby, M.D.,[1] Atul Anand, M.D.,[1] Anoop S.V. Shah, M.D.,[1, 3] Nicholas L. Mills, M.D.[1, 2] *on behalf of the High-STEACS Investigators*

[1] British Heart Foundation (BHF) Centre for Cardiovascular Science, University of Edinburgh, Edinburgh, UK.
[2] Usher Institute, University of Edinburgh, Edinburgh, UK.
[3] London School of Hygiene and Tropical Medicine, London, UK.

* contributed equally

Corresponding Author:
Professor Nicholas L Mills
BHF/University Centre for Cardiovascular Science
The University of Edinburgh
Edinburgh EH16 4SA
United Kingdom
Telephone: 0044 131 242 6515
E-mail: nick.mills@ed.ac.uk

Supplementary Text : 1

Supplementary Tables : 5

Supplementary Figures : 4

eText 1. Summary of procedures in the High-STEACS trial.

Randomisation

Block randomisation was used with sites paired based on the expected number of presentations and one site randomised to early implementation and the other to late implementation. For pragmatic reasons (shared lab facilities out of hours), the Vale of Leven and Royal Alexandra Hospital, Paisley were grouped and randomised together. This enabled implementation of the high-sensitivity assay to occur on the same date at both sites and allowed the same lab processes to be followed at both sites. The randomisation sequence was generated by a programmer at the Edinburgh Clinical Trials Unit who was not otherwise involved in the study using computer generated pseudo-random numbers.

Schematic of the High-STEACS trial design

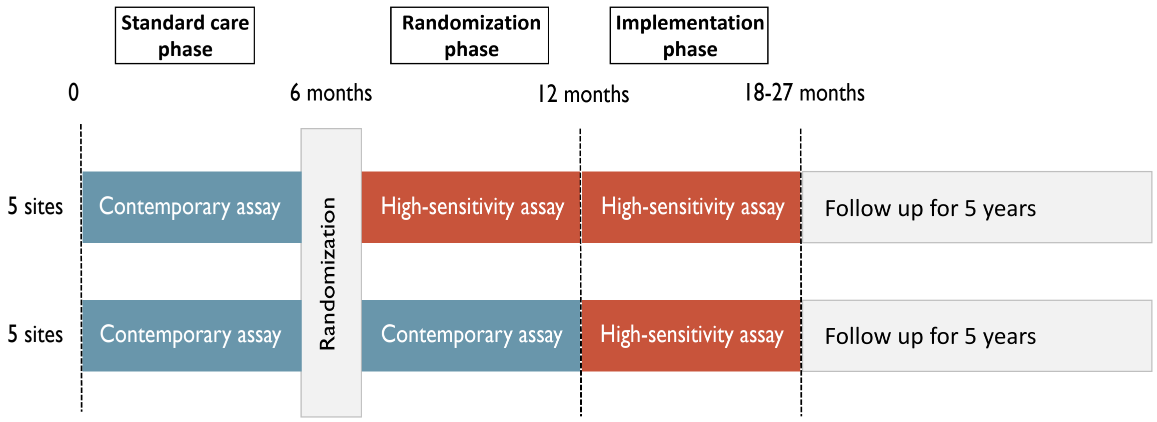

Implementation support

To support implementation, we provided written educational material and presentations at each site, training for clinical and laboratory staff, and we updated the electronic patient record to highlight the change in assay and diagnostic thresholds. Educational material on the new assay, decision thresholds and diagnosis of myocardial infarction was presented at each Emergency Department handover (twice daily) during the implementation phase to ensure wide coverage

of staff on all shift patterns. This was reinforced by specialist chest pain nurses who received detailed training prior to implementation and who support Emergency Department clinicians in the assessment of patients with suspected acute coronary syndrome.

Key details from the educational presentation formed a one-page reference guide that was posted within each department and online in the hospital guidelines portal. This included guidance for diagnosis as per the universal definition of myocardial infarction. We encouraged clinicians to select only patients with suspected acute coronary syndrome for testing, and to consider etiology, including a detailed summary of the range of cardiac and non-cardiac conditions which may be responsible for myocardial injury. This information was also presented to the wider hospital teams in medical grand round presentations prior to implementation and circulated to all general practitioners.

Every high-sensitivity cardiac troponin result reported in the electronic health record during the implementation phase was accompanied with guidance notes outlining the new assay, reporting units and thresholds. Laboratory staff also received training to ensure any queries directed to the laboratory were dealt with consistently. Finally, the research team included senior cardiologists, emergency physicians, and cardiology nurses who are clinically active within each of the hospital clusters; education was therefore reinforced at a local level by these clinical leaders throughout the implementation phase.

Adjudication of cardiac and non-cardiac causes of non-ischaemic myocardial injury

In addition to adjudicating the index diagnosis in all patients with hs-cTnI concentrations above the sex-specific 99th centile, in those with non-ischaemic myocardial injury the underlying cause was recorded prospectively and stratified as either cardiac or non-cardiac according to a

list of prespecified conditions. Cardiac diagnoses included acute heart failure, chronic heart failure, hypertensive heart disease, cardiomyopathy, takotsubo cardiomyopathy, valvular heart disease, myopericarditis, recent myocardial infarction, arrhythmia, or acute aortic dissection, whereas non-cardiac diagnoses included acute kidney injury, gastrointestinal bleed, pulmonary embolism, sepsis, chronic kidney disease, chronic obstructive pulmonary disease, and all other conditions.

Outcomes

All in-hospital and community deaths, and all hospital admissions are recorded on the Register of Deaths in Scotland and the Scottish Morbidity Record (SMR), respectively. It is a statutory requirement that any deaths occurring in Scotland, or outwith Scotland but within the United Kingdom are entered on the Register of Deaths in Scotland within eight days of death. As such, this registry is 100% complete for the study population, which was restricted to those who reside in Scotland. This assumes that patients did not emigrate in the year following enrolment. However, the Scottish population is very stable, with low levels of emigration outwith the United Kingdom.

The primary outcome was myocardial infarction or all-cause death at 5 years. Secondary outcomes include myocardial infarction, coronary revascularisation, cardiovascular death, cardiac death, all-cause death, hospitalisation for heart failure, ischaemic stroke, and major haemorrhage. International Classification of Disease (ICD)-10 codes were used to define myocardial infarction (I21, I22), heart failure (I50), ischaemic stroke (I63, I65, or I66), cardiovascular death (I00-I99) and cardiac death (I05-I09, I20-I25, or I30-I51). Bleeding was defined according to the Bleeding Academic Research Consortium (BARC) definition using

ICD-10 and Office of Population Censuses and Surveys (OPCS) codes to classify each bleeding event. Major haemorrhage was defined as BARC type 3 or type 5.

Data Sharing

The High-STEACS trial makes use of multiple routine electronic health care data sources that are linked, deidentified and held in our national safe haven, which is accessible by approved individuals who have undertaken the necessary governance training. Summary data can be made available upon request to the corresponding author (email: nick.mills@ed.ac.uk).

eTable 1. Baseline characteristics of trial participants stratified by index diagnosis

	No myocardial injury		Non-ischaemic myocardial injury		Type 1 myocardial infarction		Type 2 myocardial infarction	
	**Standard care**	**Implementation**	**Standard care**	**Implementation**	**Standard care**	**Implementation**	**Standard care**	**Implementation**
No. of participants	14,862	23,060	1,118	1,692	1,807	3,221	462	798
Age, years	59 (47, 73)	57 (46, 71)	79 (68, 85)	78 (67, 85)	69 (58, 80)	68 (57, 78)	77 (67, 84)	77 (66, 84)
Women	7,042 (47%)	10,529 (46%)	670 (60)	1,003 (59)	748 (41)	1,262 (39)	275 (60)	425 (53)
**Presenting complaint***								
Chest pain	8,677 (83%)	19,414 (84%)	315 (40)	722 (43)	1,238 (89)	2,869 (89)	257 (72)	569 (71)
Dyspnea	383 (4%)	724 (3%)	196 (25)	394 (23)	64 (5)	107 (3)	33 (9)	100 (13)
Palpitation	291 (3%)	700 (3%)	34 (4)	99 (6)	6 (0)	12 (0)	32 (9)	41 (5)
Syncope	567 (5%)	1,242 (5%)	151 (19)	256 (15)	30 (2)	72 (2)	18 (5)	32 (4)
Other	490 (5%)	968 (4%)	101 (13)	221 (13)	60 (4)	161 (5)	16 (4)	56 (7)
**Past medical history**								
Myocardial infarction	1,353 (9%)	1,482 (6%)	143 (13)	201 (12)	301 (17)	384 (12)	73 (16)	110 (14)
Cerebrovascular disease	877 (6%)	1,038 (5%)	154 (14)	186 (11)	132 (7)	236 (7)	63 (14)	91 (11)
Diabetes mellitus	961 (6%)	1,079 (5%)	155 (14)	193 (11)	302 (17)	507 (16)	66 (14)	105 (13)
**Previous revascularisation**								
PCI	1,204 (8%)	1,540 (7%)	76 (7)	135 (8)	198 (11)	315 (10)	42 (9)	63 (8)
CABG	238 (2%)	296 (1%)	28 (3)	45 (3)	47 (3)	59 (2)	16 (3)	22 (3)
**Medications at presentation**								
Aspirin	4,231 (28%)	5,231 (23%)	433 (39)	567 (34)	682 (38)	1,042 (32)	211 (46)	318 (40)
Dual anti-platelet therapy†	583 (4%)	520 (2%)	66 (6)	66 (4)	124 (7)	117 (4)	30 (6)	39 (5)
Statin	5,994 (40%)	8,112 (35%)	607 (54)	840 (50)	906 (50)	1,503 (47)	272 (59)	447 (56)
ACE inhibitor or ARB	4,681 (31%)	6,604 (29%)	467 (42)	735 (43)	755 (42)	1,265 (39)	238 (52)	340 (43)
Beta-blocker	4,183 (28%)	5,383 (23%)	402 (36)	564 (33)	648 (36)	974 (30)	227 (49)	316 (40)
Oral anti-coagulant‡	938 (6%)	1,220 (5%)	161 (14)	238 (14)	112 (6)	181 (6)	82 (18)	113 (14)

**Electrocardiogram§**								
Normal			254 (36)	472 (36)	582 (39)	1,015 (35)	82 (20)	152 (21)
Myocardial ischaemia			54 (8)	97 (7)	566 (38)	1,325 (46)	134 (32)	279 (38)
ST-segment elevation			19 (3)	53 (4)	232 (16)	647 (22)	9 (2)	29 (4)
ST-segment depression			49 (7)	59 (5)	281 (19)	592 (20)	109 (26)	202 (27)
T-wave inversion			101 (14)	147 (11)	282 (19)	505 (17)	74 (18)	118 (16)
**Physiological parameters**								
Heart rate, beats per minute			87 (71, 105)	86 (70, 106)	77 (67, 91)	76 (64, 90)	94 (75, 124)	99 (78, 124)
Systolic blood pressure, mmHg			133 (117, 154)	135 (117, 154)	140 (122, 158)	141 (124, 160)	132 (111, 151)	130 (113, 152)
**Haematology and clinical chemistry**								
Haemoglobin, g/L	138 (127, 149)	139 (128, 150)	129 (113, 143)	129 (115, 143)	137 (122, 149)	139 (125, 151)	129 (110, 145)	132 (115, 146)
eGFR, mL/min	95 (81, 107)	96 (82, 107)	68 (44, 85)	68 (45, 86)	82 (61, 95)	82 (61, 95)	70 (48, 86)	69 (48, 87)
Peak hs-cTnI, ng/L	3 (1, 6)	3 (1, 6)	65 (35, 222)	62 (36, 197)	528 (84, 4,434)	1,117 (124, 8,411)	102 (47, 551)	125 (47, 523)

Presented as No. (%), median (inter-quartile range).

Abbreviations: ACE = angiotensin converting enzyme; ARB = angiotensin receptor blockers; eGFR = estimated glomerular filtration rate; CABG = coronary artery bypass grafting; PCI = percutaneous coronary intervention. *Presenting symptom was missing in 4,466 (12%) patients. †Two medications from aspirin, clopidogrel, prasugrel or ticagrelor. ‡Includes warfarin or direct oral anticoagulants. §Electrocardiographic findings and physiological parameters only reported for those with elevation in cardiac troponin concentrations.

eTable 2. Management during index hospital admission stratified by peak troponin concentration and study phase

	No myocardial injury		Myocardial injury or infarction			
			Reclassified by hs-cTnI assay		Identified by cTnI assay	
	**Standard care**	**Implementation**	**Standard care**	**Implementation**	**Standard care**	**Implementation**
No. of participants	14,862 (39)	23,060 (61)	720 (41)	1,051 (59)	3,396 (40)	5,193 (60)
Duration of stay, hrs	7 (3, 24)	4 (3, 20)	21 (4, 100)	51 (20, 133)	82 (19, 186)	78 (37, 164)
Coronary angiography*	204 (1)	329 (1)	29 (4)	111 (11)	1,108 (33)	2,177 (42)
PCI/CABG	112 (1)	187 (1)	23 (3)	51 (5)	706 (21)	1,535 (30)
New anti-platelet agent	795 (5)	976 (4)	64 (9)	194 (18)	1,408 (41)	2,428 (47)
New dual anti-platelet therapy†	248 (2)	336 (1)	35 (5)	124 (12)	1,144 (34)	2,080 (40)
New statin therapy	419 (3)	608 (3)	32 (4)	79 (8)	660 (19)	1,263 (24)
New ACE inhibitor or ARB	287 (2)	479 (2)	34 (5)	77 (7)	671 (20)	1,163 (22)
New beta-blocker	765 (5)	1,092 (5)	65 (9)	164 (16)	828 (24)	1,502 (29)

Presented as No. (%), median (inter-quartile range).

* Angiography and revascularisation within 30 days of presentation

† Two medications from aspirin, clopidogrel, prasugrel or ticagrelor

eTable 3. Management during index hospital admission stratified by diagnosis and study phase

	**No myocardial injury**		**Non-ischaemic myocardial injury**		**Type 1 myocardial infarction**		**Type 2 myocardial infarction**	
	**Standard care**	**Implementation**	**Standard care**	**Implementation**	**Standard care**	**Implementation**	**Standard care**	**Implementation**
No. of participants	14,862	23,060	1,118	1,692	1,807	3,221	462	798
Duration of stay, hrs	7 (3, 24)	4 (3, 20)	128 (27, 305)	103 (28, 263)	75 (26, 134)	74 (46, 124)	109 (47, 233)	94 (37, 199)
Coronary angiography*	204 (1)	329 (1)	38 (3)	85 (5)	1,024 (57)	2,093 (65)	38 (8)	83 (10)
PCI/CABG	112 (1)	187 (1)	6 (1)	<5 (0)	700 (39)	1,553 (48)	6 (1)	14 (2)
New anti-platelet agent	795 (5)	976 (4)	130 (12)	196 (12)	1,160 (64)	2,232 (69)	80 (17)	140 (18)
New dual anti-platelet therapy†	248 (2)	336 (1)	64 (6)	87 (5)	1,000 (55)	2,003 (62)	47 (10)	78 (10)
New statin therapy	419 (3)	608 (3)	51 (5)	76 (4)	577 (32)	1,194 (37)	29 (6)	47 (6)
New ACE inhibitor or ARB	287 (2)	479 (2)	64 (6)	112 (7)	566 (31)	1,027 (32)	37 (8)	71 (9)
New beta-blocker	765 (5)	1,092 (5)	106 (9)	198 (12)	645 (36)	1,242 (39)	67 (15)	171 (21)

Presented as No. (%), median (inter-quartile range).

* Angiography and revascularisation within 30 days of presentation

† Two medications from aspirin, clopidogrel, prasugrel or ticagrelor

eTable 4. Cox-proportional hazards model for subsequent myocardial infarction or all-cause death at 5 years stratified by index diagnosis

	**Adjusted hazard ratios**
**UDMI 3**	
Type 1	0·92 [0·83-1·01]
Type 2	0·98 [0·84-1·14]
Non-ischaemic myocardial injury	0·83 [0·75-0·91]
**UDMI 4**	
Type 1	0·92 [0·84-1·01]
Type 2	0·95 [0·80-1·11]
Non-ischaemic myocardial injury	0·85 [0·77-0·94]

* Cox-proportional hazards model adjusting for hospital site as random effect, season, age, sex, diabetes mellitus, previous myocardial infarction and previous cerebrovascular disease

Abbreviations: UDMI= universal definition of myocardial infarction

eTable 5. Outcomes after 5 years in participants stratified by index diagnosis

	No myocardial injury		Non-ischaemic myocardial injury		Type 1 myocardial infarction		Type 2 myocardial infarction	
	**Standard care**	**Implementation**	**Standard care**	**Implementation**	**Standard care**	**Implementation**	**Standard care**	**Implementation**
No. of participants	14,862	23,060	1,118	1,692	1,807	3,221	462	798
**Myocardial infarction or all-cause death**	3,194 (21)	4,361 (19)	804 (72)	1,064 (63)	827 (46)	1,342 (42)	268 (58)	457 (57)
**Myocardial infarction**	584 (4)	757 (3)	108 (10)	108 (6)	333 (18)	526 (16)	46 (10)	74 (9)
**Coronary revascularisation**	729 (5)	808 (4)	37 (3)	43 (3)	200 (11)	271 (8)	16 (3)	32 (4)
**All-cause death**	2,804 (19)	3,837 (17)	765 (68)	1,023 (60)	663 (37)	1,040 (32)	246 (53)	432 (54)
**Death from cardiovascular causes**	841 (6)	1,186 (5)	322 (29)	447 (26)	356 (20)	592 (18)	108 (23)	212 (27)
**Death from cardiac causes**	569 (4)	803 (3)	229 (20)	333 (20)	309 (17)	498 (15)	89 (19)	168 (21)
**Hospital admission for heart failure**	744 (5)	931 (4)	213 (19)	305 (18)	256 (14)	381 (12)	112 (24)	178 (22)
**Ischaemic stroke**	271 (2)	351 (2)	51 (5)	59 (3)	52 (3)	94 (3)	15 (3)	27 (3)
**Major haemorrhage***	220 (1)	260 (1)	23 (2)	39 (2)	43 (2)	49 (2)	11 (2)	22 (3)

Presented as No. (%). *Defined as Bleeding Academic Research Consortium type 3 or 5

eFigure 1. CONSORT Diagram of the Trial and Populations

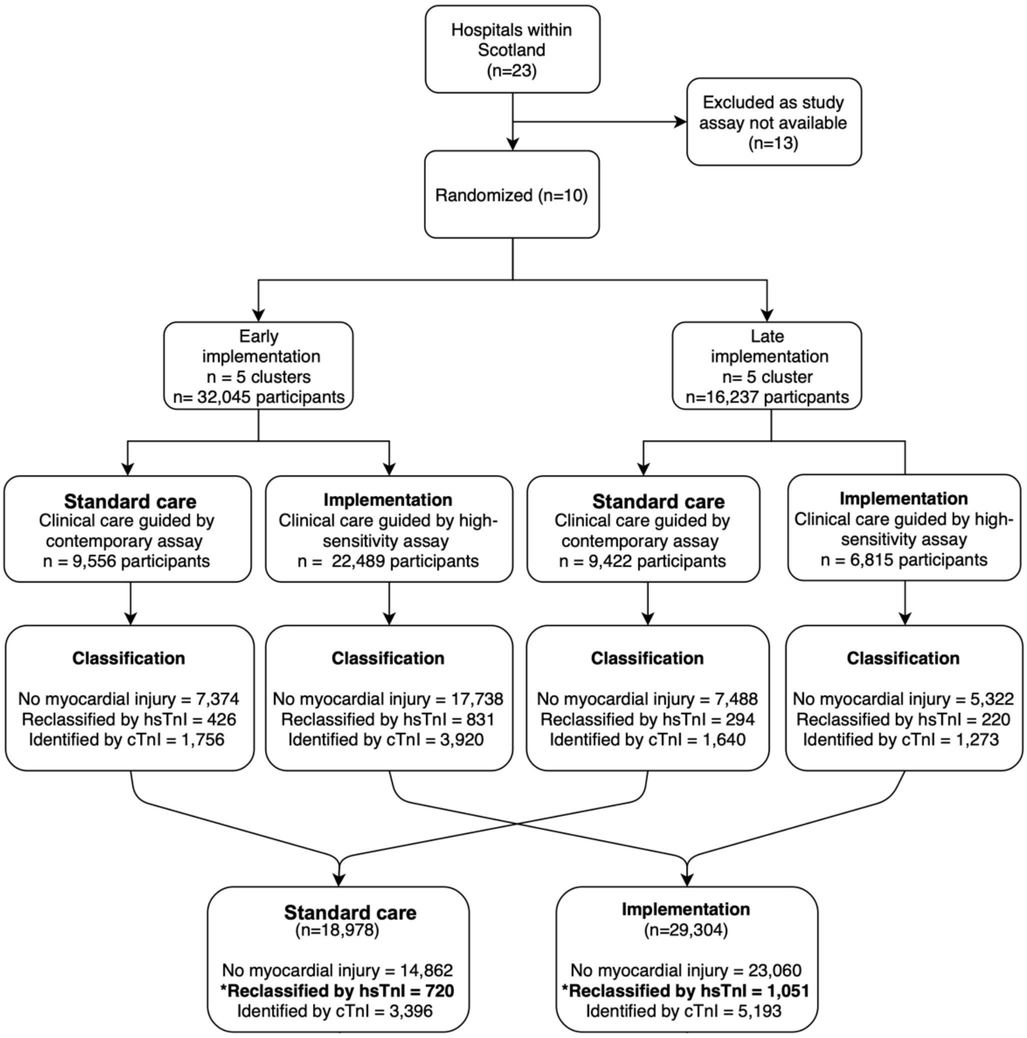

eFigure 2. Outcomes at 5 years in patients reclassified by the high-sensitivity cardiac troponin I assay before and after implementation

**Primary outcome**	**Standard care n (%)**	**Implementation n (%)**	**Adjusted hazard ratio 95% CI**
Myocardial infarction or all-cause death	456 (63.3%)	567 (53.9%)	0.82 (0.72-0.94)
**Secondary outcomes**			
Myocardial infarction	103 (14.3%)	113 (10.8%)	0.78 (0.59-1.03)
Unplanned revascularisation	41 (5.7%)	45 (4.3%)	0.73 (0.46-1.16)
All-cause death	416 (57.8%)	501 (47.7%)	0.80 (0.69-0.92)
Cardiovascular death	170 (23.6%)	222 (21.1%)	0.86 (0.70-1.06)
Cardiac death	118 (16.4%)	167 (15.9%)	0.92 (0.72-1.18)
Hospital admission with heart failure	144 (20.0%)	198 (18.8%)	0.97 (0.78-1.21)
Ischaemic stroke	34 (4.7%)	38 (3.6%)	0.81 (0.51-1.27)

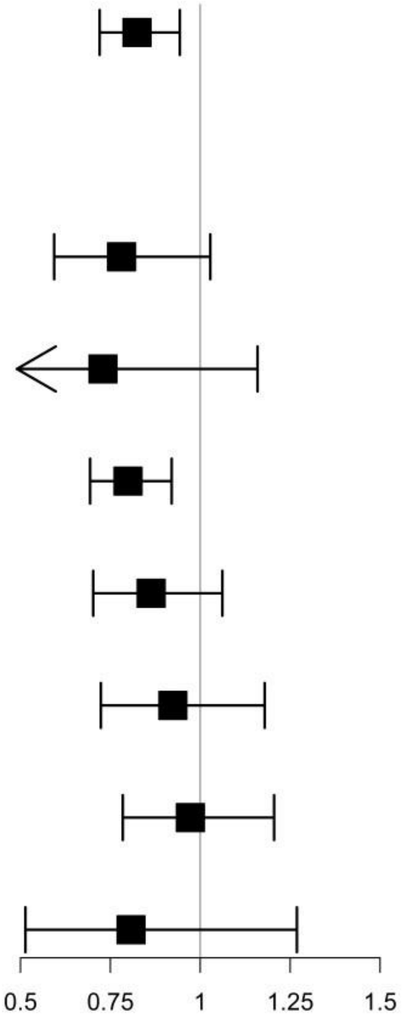

eFigure 3. Outcomes at 5 years before and after implementation stratified by index diagnosis

A) Outcomes in patients with an index diagnosis of type 1 myocardial infarction.

Primary outcome	Standard care n (%)	Implementation n (%)	Adjusted hazard ratio 95% CI
Myocardial infarction or all-cause death	827 (45.8%)	1342 (41.7%)	0.92 (0.83-1.01)
**Secondary outcomes**			
Myocardial infarction	333 (18.4%)	526 (16.3%)	0.93 (0.80-1.07)
Unplanned revascularisation	200 (11.1%)	271 (8.4%)	0.75 (0.62-0.90)
All-cause death	663 (36.7%)	1040 (32.3%)	0.90 (0.81-1.00)
Cardiovascular death	356 (19.7%)	592 (18.4%)	0.96 (0.84-1.10)
Cardiac death	309 (17.1%)	498 (15.5%)	0.92 (0.80-1.07)
Hospital admission with heart failure	256 (14.2%)	381 (11.8%)	0.92 (0.78-1.08)
Ischaemic stroke	52 (2.9%)	94 (2.9%)	0.99 (0.70-1.41)

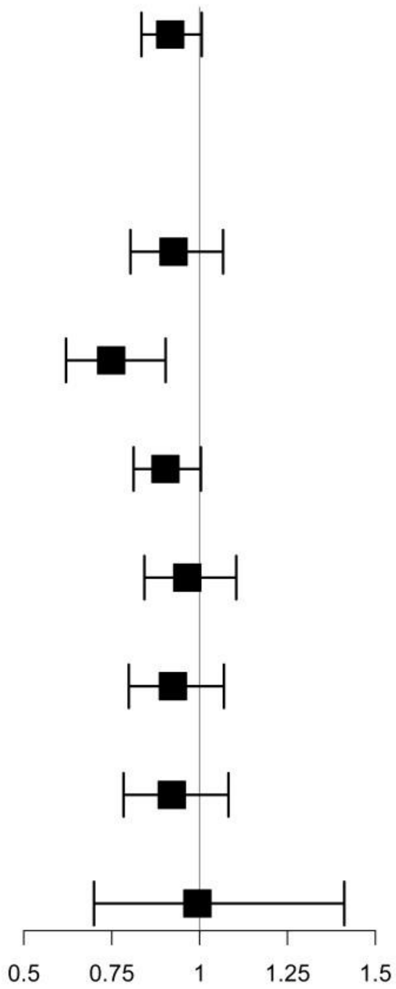

B) Outcomes in patients with an index diagnosis of type 2 myocardial infarction.

Primary outcome	Standard care n (%)	Implementation n (%)	Adjusted hazard ratio 95% CI
Myocardial infarction or all-cause death	268 (58.0%)	457 (57.3%)	0.98 (0.84-1.14)
**Secondary outcomes**			
Myocardial infarction	46 (10.0%)	74 (9.3%)	0.92 (0.63-1.33)
Unplanned revascularisation	16 (3.5%)	32 (4.0%)	1.17 (0.65-2.10)
All-cause death	246 (53.2%)	432 (54.1%)	1.03 (0.87-1.21)
Cardiovascular death	108 (23.4%)	212 (26.6%)	1.16 (0.91-1.46)
Cardiac death	89 (19.3%)	168 (21.1%)	1.10 (0.84-1.43)
Hospital admission with heart failure	112 (24.2%)	178 (22.3%)	0.95 (0.75-1.20)
Ischaemic stroke	15 (3.2%)	27 (3.4%)	1.00 (0.51-1.99)

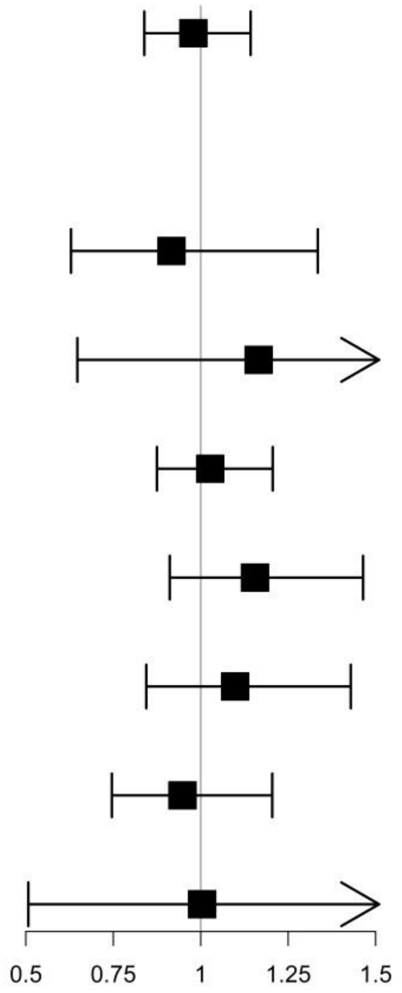

C) Outcomes in patients with an index diagnosis of non-ischaemic myocardial injury.

Primary outcome	Standard care n (%)	Implementation n (%)	Adjusted hazard ratio 95% CI
Myocardial infarction or all-cause death	804 (71.9%)	1064 (62.9%)	0.83 (0.75-0.91)
**Secondary outcomes**			
Myocardial infarction	108 (9.7%)	108 (6.4%)	0.66 (0.51-0.87)
Unplanned revascularisation	37 (3.3%)	43 (2.5%)	0.76 (0.49-1.20)
All-cause death	765 (68.4%)	1023 (60.5%)	0.85 (0.77-0.94)
Cardiovascular death	322 (28.8%)	447 (26.4%)	0.84 (0.71-0.99)
Cardiac death	229 (20.5%)	333 (19.7%)	0.89 (0.73-1.08)
Hospital admission with heart failure	213 (19.1%)	305 (18.0%)	0.95 (0.80-1.13)
Ischaemic stroke	51 (4.6%)	59 (3.5%)	0.75 (0.52-1.07)

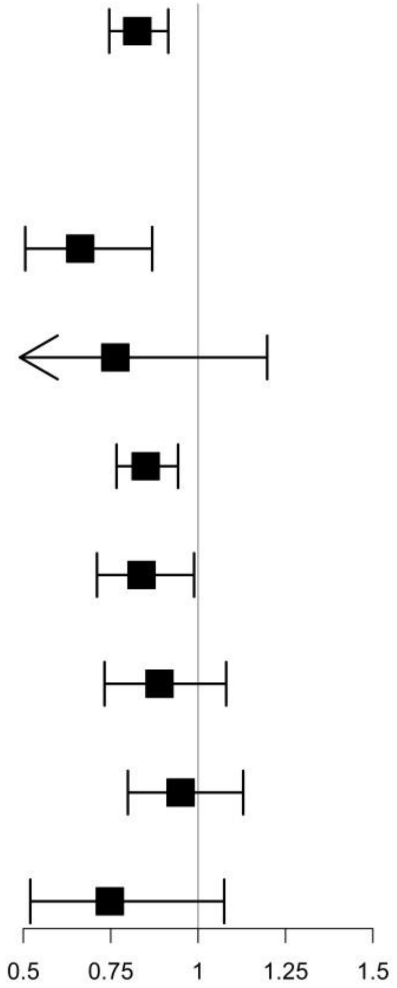

eFigure 4. Outcomes at 5 years before and after implementation stratified by cause of non-ischaemic myocardial injury

A) Outcomes in patients with non-ischaemic myocardial injury due to a cardiac cause

Primary outcome	Standard care n (%)	Implementation n (%)	Adjusted hazard ratio 95% CI
Myocardial infarction or all-cause death	322 (66.0%)	450 (53.1%)	0.69 (0.60-0.80)
**Secondary outcomes**			
Myocardial infarction	63 (12.9%)	57 (6.7%)	0.49 (0.34-0.72)
Unplanned revascularisation	15 (3.1%)	25 (3.0%)	1.01 (0.52-1.96)
All-cause death	297 (60.9%)	429 (50.6%)	0.76 (0.66-0.89)
Cardiovascular death	182 (37.3%)	237 (28.0%)	0.69 (0.56-0.84)
Cardiac death	149 (30.5%)	203 (24.0%)	0.73 (0.58-0.91)
Hospital admission with heart failure	144 (29.5%)	194 (22.9%)	0.79 (0.64-0.97)
Ischaemic stroke	19 (3.9%)	34 (4.0%)	0.94 (0.55-1.62)

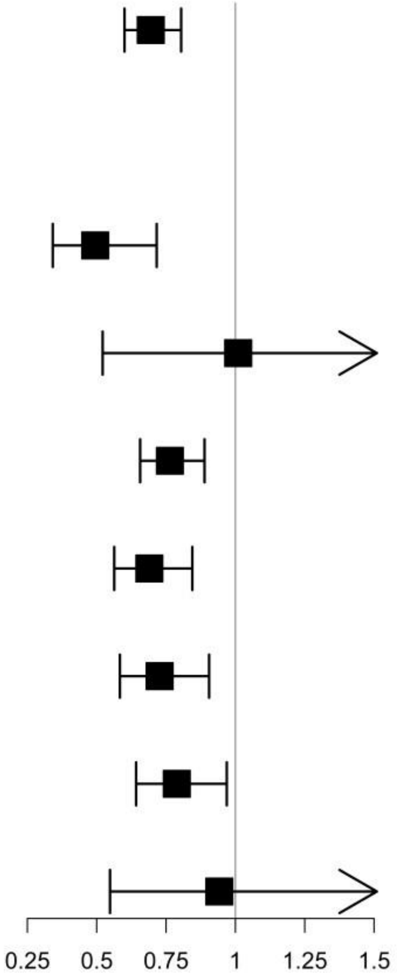

B) Outcomes in patients with non-ischaemic myocardial injury due to a non-cardiac cause

Primary outcome	Standard care n (%)	Implementation n (%)	Adjusted hazard ratio 95% CI
Myocardial infarction or all-cause death	479 (76.5%)	605 (72.8%)	0.95 (0.83-1.09)
**Secondary outcomes**			
Myocardial infarction	45 (7.2%)	49 (5.9%)	0.85 (0.56-1.28)
Unplanned revascularisation	22 (3.5%)	17 (2.0%)	0.58 (0.30-1.12)
All-cause death	465 (74.3%)	586 (70.5%)	0.94 (0.82-1.07)
Cardiovascular death	139 (22.2%)	206 (24.8%)	1.06 (0.83-1.35)
Cardiac death	79 (12.6%)	126 (15.2%)	1.08 (0.78-1.50)
Hospital admission with heart failure	69 (11.0%)	107 (12.9%)	1.17 (0.86-1.59)
Ischaemic stroke	32 (5.1%)	24 (2.9%)	0.54 (0.31-0.92)

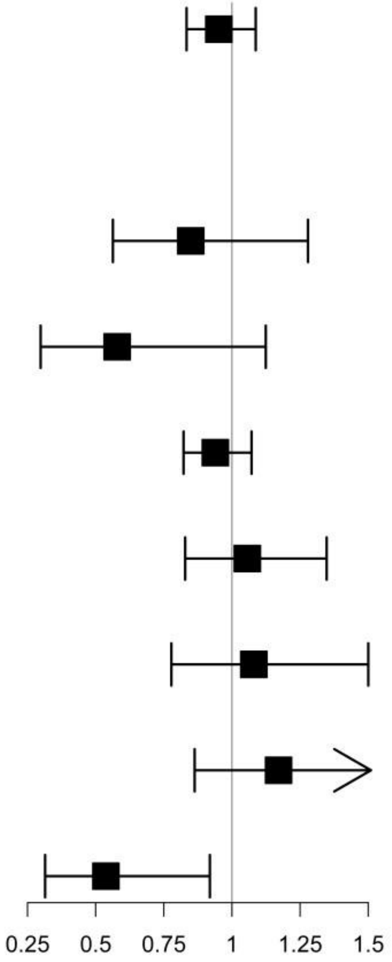

Supplement: Supplementary file 1 — Supplementary information: Supplementary eText 1, eTables 1-5, and eFigures 1-4 [file leek075009.ww.pdf]
